# Three-Dimensional Analysis of the Curvature of the Femoral Canal in 426 Chinese Femurs

**DOI:** 10.1155/2015/318391

**Published:** 2015-10-28

**Authors:** Xiu-Yun Su, Zhe Zhao, Jing-Xin Zhao, Li-Cheng Zhang, An-Hua Long, Li-Hai Zhang, Pei-Fu Tang

**Affiliations:** ^1^Department of Orthopaedics, Chinese PLA General Hospital, No. 28 Fuxing Road, Beijing 100853, China; ^2^Department of Orthopaedics, Affiliated Hospital of the Academy of Military Medical Sciences, No. 8 Dongdajie Road, Beijing 100071, China; ^3^Department of Orthopaedics, Beijing Tsinghua Chang Gung Hospital, No. 1 Block Tiantongyuan North, Beijing 102218, China

## Abstract

*Purpose*. The human femur has long been considered to have an anatomical anterior curvature in the sagittal plane. We established a new method to evaluate the femoral curvature in three-dimensional (3D) space and reveal its influencing factors in Chinese population. *Methods*. 3D models of 426 femurs and the medullary canal were constructed using Mimics software. We standardized the positions of all femurs using 3ds Max software. After measuring the anatomical parameters, including the radius of femoral curvature (RFC) and banking angle, of the femurs using the established femur-specific coordinate system, we analyzed and determined the relationships between the anatomical parameters of the femur and the general characteristics of the population. *Results*. Pearson's correlation analyses showed that there were positive correlations between the RFC and height (*r* = 0.339, *p* < 0.001) and the femoral length and RFC (*r* = 0.369, *p* < 0.001) and a negative correlation between the femoral length and banking angle (*r* = −0.223, *p* < 0.001). Stepwise linear regression analyses showed that the most relevant factors for the RFC and banking angle were the femoral length and gender, respectively. *Conclusions*. This study concluded that the banking angle of the femur was significantly larger in female than in male.

## 1. Introduction

The anterior curvature has been regarded as an important anatomical characteristic of the femur and has been extensively studied by anthropologists [1–4]. Due to its important implications for intramedullary (IM) nailing and total knee arthroplasty (TKA), orthopedic surgeons pay close attention to anterior femoral curvature. Although IM nailing and TKA constitute effective treatments for proximal femur or femoral shaft fractures and knee osteoarthritis and have aided in the design of femoral implants by manufacturers [5, 6], a series of complications caused by mismatch between the curvatures of the femur and implant have been reported, including nail impingement against the anterior or lateral cortex [7, 8], the anterior cortex encroachment or penetration [9–12], and the anterior cortex fracture [13].

Previous studies have proposed different measurement methods for the anterior curvature of the femur. Although the traditional measurement method uses caliper to measure the bone on the osteometric board [1, 2, 14], the most common measurement methods used by medical researchers are based on lateral radiographs or images of the femur. Some authors have calculated and measured the femoral curvature from the outer surfaces of the femurs [15, 16], while other researchers have calculated the curvature of the femoral medullary canal based on lateral radiographs [13, 17]. Furthermore, with the rapid development of computer-aided design (CAD) software and its applications in the digital medical imaging process, some authors have simulated or reconstructed lateral radiographs or images using computed tomography- (CT-) derived three-dimensional (3D) femoral models and subsequently obtained measurements of the femoral curvature using these models [6, 7, 18, 19].

All of these previously described measurement methods are based on an a priori acceptance that the plane, in which the femoral curvature is located, was precisely in parallel with the sagittal plane. However, several studies have documented the existence of medial or lateral bowing of the femur on the coronal plane using different measurement methods with anteroposterior radiographs [20–23], a phenomenon that is closely correlated with age [20], the osteoporosis femur fracture [23], and the revision TKA [21].

Thus, neither the anterior curvature nor the lateral bow can provide an accurate description and detailed knowledge of the femoral curvature in 3D space. For the first time, Chantarapanich et al. reconstructed and calculated the femoral curvature in 3D space and concluded that the femoral 3D curvature was closely correlated with the curvature on the sagittal plane, irrespective of that on the coronal plane [24]. In this study, the authors tried to reveal the impact of the femoral 3D curvature on the anterior and coronal curvatures. The hypothesis was that there might be a potentially ignored factor influencing the relationship among the femoral 3D curvature, anterior curvature, and coronal curvature.

In the present study, the first objective was to establish a new method to calculate the anatomical parameters of the femoral curvature in 3D space. The second objective was to determine the relationships between the 3D femoral curvature and the individual's height, sex, age, and femoral length in a large group of Chinese subjects.

## 2. Materials and Methods

This study was a retrospective medical imaging investigation, which has been approved by the Ethics Committee of the Chinese PLA General Hospital (code: S2014-035-01). Due to its retrospective nature and the fact that the patient data were anonymous, a waiver of patients' informed consent was granted. We collected CT data from a total of 426 femurs from 213 consecutive patients who underwent lower-extremity CT between December 2009 and December 2012 at our institution. A part of patients was included in our previous studies [25, 26]. All CT scans were performed using the SOMATOM sensation open CT system (Siemens AG, Erlangen, Germany) with slice thicknesses of 1.2 mm. Patients with evidence of lower-extremity trauma or deformity were excluded. Demographic data, including age, sex, height, and weight, were obtained from electronic medical records.

### 2.1. D Reconstruction of the Femur and Its Canal

**Figure 1:**
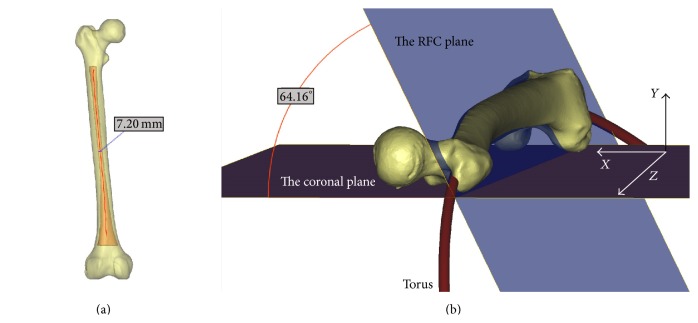
Three-dimensional models of the entire femur and its canal. The centerline of the medullary canal was established, and the isthmic diameter was then calculated as 7.20 mm (a). A circle was fitted into the centerline of the medullary canal, and a torus was then created using the fitted circle and isthmic diameter (b). The banking angle was defined as the angle between the RFC plane, in which the fitted circle was located, and the coronal plane, on which the femoral model was placed; this angle was measured at 64.16 degrees.

CT data in the Digital Imaging and Communications in Medicine (DICOM) format were imported into an interactive medical image control system (Mimics, Materialise NV, Leuven, Belgium) to reconstruct 3D models of the whole femur and its medullary canal from the lesser trochanter to the flare of the condyles. The 3D femoral model and 3D femoral canal model were created in the Stereo-Lithography (STL) format (Figure 1(a)).

Using Mimics software, a centerline was automatically calculated to fit the femoral canal model with default parameters. The centerline extraction produced the center of the femoral canal without the endings, with the centerline deviating with the contour of the femoral canal. The endings were cut with a centerline-ending tool to create an adequate femoral canal centerline. Three continuing inscribed circles at each control point on the centerline with the smallest diameters were selected, of which the smallest circle was considered as the femoral narrowest isthmic and the corresponding diameter as the isthmic diameter (Figure 1(a)).

**Figure 2:**
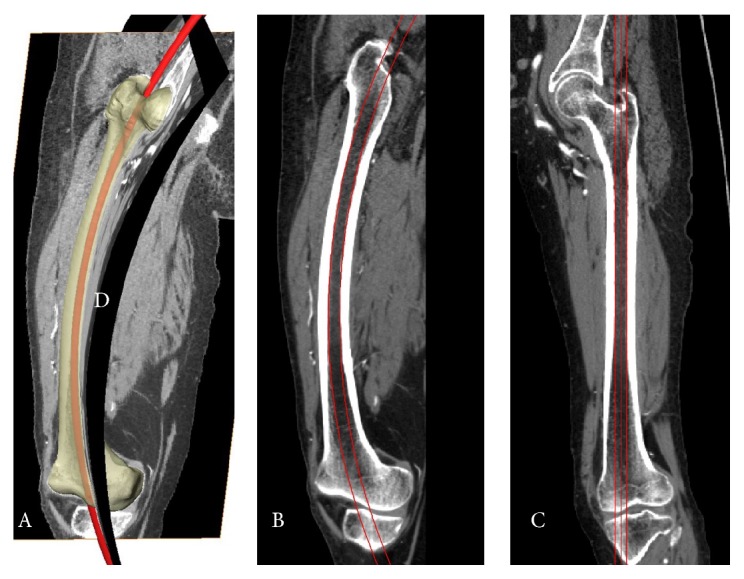
The simulation experiment to verify the interaction between the torus and inner femoral cortex. A torus was created using the canal's centerline and femoral isthmic diameter. Three orthogonal planes were created. The first curved plane (plane D) was part of a cylinder surface (left side), which was perpendicular to the RFC plane (plane A or B). The axial plane (shown in the video of Supplementary Material) was perpendicular to plane (D) and plane (A) or (B), simultaneously, and moved along the canal's centerline in the video recording. After reformatting the CT scan along the canal's centerline, the femoral canal became almost straight along plane (C), which was created after stretching plane (D).

The 3D whole femoral model, 3D femoral canal model, and femoral canal centerline were subsequently exported to undergo engineering modification in 3-Matic software (Materialise). Using our established method, the femoral canal centerline could be fitted to a circle. According to Bruns et al. [14], the femoral curvature could be considered as a part of a virtual circle, the radius of which could be considered as the radius of the femoral curvature (RFC). Thus, the radius of the previous fitted circle from the femoral canal centerline could be considered the RFC. A torus, representing an IM nail, was created using the circle as the centerline and the femoral isthmic diameter as the diameter. The torus in STL format was exported to Mimics to determine if there was any interaction between the torus and the inner femoral cortex in three orthogonal cross planes (Figure 2 and S1 video (in Supplementary Material available online at http://dx.doi.org/10.1155/2015/318391)).

### 2.2. Construction of Femur-Specific Coordinate System

We constructed the patient-specific anatomical coordinate system on the basis of femoral geometry. Although each femoral model had a CT-based coordinate system, the position of the femur while performing CT scans influenced the direction of the CT-based coordinate system [28]. Thus, we developed a reliable method for normalizing the 3D orientation of each femoral model in 3ds Max software (Autodesk, available at http://www.autodesk.com/). The femur 3D model in STL format was imported to 3ds Max, and the coronal plane, as a substitute for an osteometric table, was created on the global Cartesian* X*-*Z* plane. The axial plane, as a substitute for the stationary end of an osteometric board, was created on the global Cartesian* X*-*Y* plane. Subsequently, we performed a physics simulation of placing the femur on an osteometric board using MassFX tools. The femoral model was set as a dynamic rigid body, and the two planes were set as a static rigid body.

We first applied gravity along the global *y*-axis. The femoral 3D object fell on the coronal plane to simulate the femur being placed on an osteometric board in the supine position. Thus, the most posterior points of the medial and lateral femoral condyles and the greater trochanter were in direct contact with the coronal plane (Figure 1(b)). Next, we applied gravity along the global *z*-axis, which constrained movement of the femur along the *z*-axis and rotation around the *x*- and *z*-axes. The 3D femoral model was placed on the axial plane to ensure that the most distal points of both femoral condyles would be in contact with the axial plane. Next, the standardized femoral 3D model in STL format was exported to the 3-Matic software for further measurements, and both the femoral canal 3D model and the centerline circle were then aligned with the standardized model using the transformation matrix between the positions of the femurs before and after standardization. The positions of all femoral models and the corresponding canals and circles were standardized using this method.

### 2.3. Calculation of the Banking Angle of the Femur

The phrase “banking angle” is derived from aviation terminology and corresponds to the angle between the aircraft's wing and the horizontal plane while the airplane is making a turn. In this study, the banking angle was considered to be the inclination angle of the femoral curvature relative to the coronal plane (Figure 1(b)), which was measured between the coronal plane and the plane in which the canal fitted circle was located. We defined the direction of the medial side of the femur as the opening direction of the banking angle to be measured.

The femoral length was defined as the distance from the most superior point of the femoral head to the most inferior point of the distal condyles, which could be acquired from the property information of the femoral 3D model.

### 2.4. The RFC Projection on the Sagittal Plane

**Figure 3:**
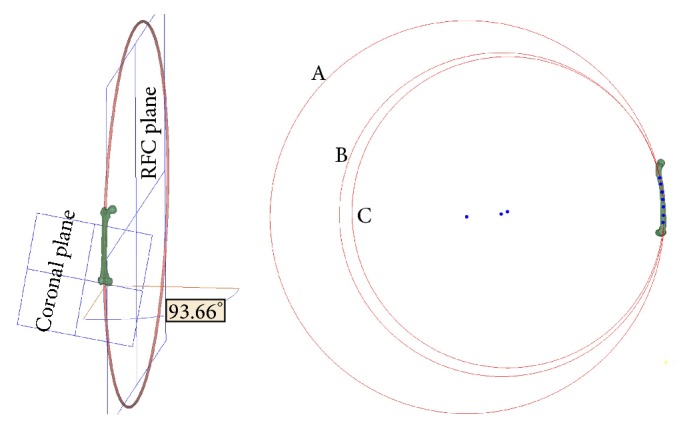
A femoral model with a banking angle of 93.66 degrees. In the sagittal plane (right side), seven points, which were distributed equally on the projected centerline of the canal, were manually selected between the lesser trochanter and the flare of the condyles, which divided the centerline of the canal into three sections, including the proximal third, middle third, and distal third. The radii of circles (A), (B), and (C) fitted from the proximal, middle, and distal sections of the canal centerline were 1,077.79, 887.93, and 852.98 mm, respectively.

**Figure 4:**
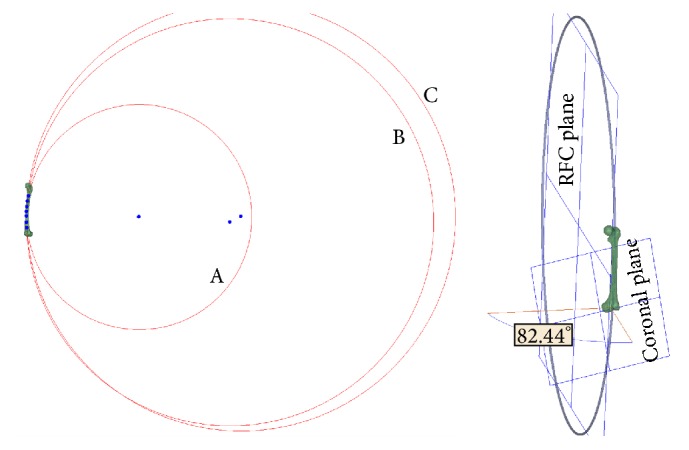
A femoral model with a banking angle of 82.44 degrees. In the sagittal plane (left side), the radii of circles (A), (B), and (C) fitted from the proximal, middle, and distal sections of the canal centerline were 882.92, 1,596.73, and 1,681.7 mm, respectively.

As the sagittal RFC was most commonly studied in previous studies, we projected the circle of the femoral canal onto the sagittal plane to understand how the sagittal RFC was altered by the 3D RFC. On the sagittal projected RFC, seven points were manually selected and distributed equally between the lesser trochanter and the flare of the condyles of the femur. This divided the sagittal RFC into three sections, including the proximal third, middle third, and distal third. On the sagittal plane, these three sections could be fitted into three corresponding circles, the radii of which could be calculated. We selected two femoral models with contrasting banking angles to perform this experiment (Figures 3 and 4).

### 2.5. Statistical Analysis

Sample-size calculations were based on our previous study of the characteristics of the femoral canal isthmus [26]. The femoral length was regarded as the primary outcome measure for sample-size calculation. Given a type I error of 5%, a power of 80%, and standard deviation of 20, it was estimated that 85 patients would be required at least in each group in order to detect a difference of 10 in the femoral length between groups (2-tailed test). Thus, the sample size of this study was enough for the required analyses. All data were imported into SPSS software for regression analysis and statistical comparison (SPSS, Chicago, IL, USA). The normal distribution of all of the data was tested using the Kolmogorov-Smirnov method. The data with nonnormal distribution were compared using the Manny-Whitney *U* test. The data with normal distribution were compared between different genders using independent samples Student's* t*-test and between different lateralities using paired samples Student's* t*-test, respectively. Correlations between variables were determined using Pearson's correlation analysis. After verification of the absence of the collinearity among the independent variables, stepwise linear regression model was applied to investigate the influential factors of the RFC and the banking angle. Statistical significance was set at *p* values of 0.05 or less.

## 3. Results

The main characteristics of the population included in this study and the anatomical parameters of the femur were summarized in Table 1. The results of independent and paired samples *t*-tests were shown in Tables 1 and 2, respectively.

The femoral length was 428.07 ± 25.38 mm. The RFC was 971.44 ± 211.68 mm, which was significantly larger in the male than in female (*p* < 0.001). The banking angle was 93.48 ± 11.95 degrees, which was significantly larger in the female than in male.

Pearson's correlation analysis showed correlations among the main characteristics of the population and the anatomical parameters of the femur (Table 3). There were positive correlations between the femoral length and height (*r* = 0.845, *p* < 0.001), between the RFC and height (*r* = 0.339, *p* < 0.001), and between the RFC and femoral length (*r* = 0.369, *p* < 0.001).

Stepwise linear regression analyses were applied with the RFC and the banking angle as the dependent variables. Using the final regression model, the RFC was calculated as 267.36 + 2.23 × the femoral length – 2.88 × the banking angle + 57.49 × the femoral laterality – 50.3 × the gender (*R* ^2^ = 0.191). Similarly, the banking angle was calculated as 69.4 + 5.7 × the gender + 0.236 × the age – 0.01 × the RFC + 0.157 × the weight (*R* ^2^ = 0.158).

A femoral model with a banking angle of 93.66 degrees is shown in Figure 3. In the sagittal plane, the radii of the circles fitted from the proximal, middle, and distal sections of the canal centerline were 1,077.79, 887.93, and 852.98 mm, respectively. The banking angle of another femur shown in Figure 4 was 82.44 degrees. The radii of the circles fitted from the proximal, middle, and distal distances were 882.92, 1,596.73, and 1,681.7 mm, respectively.

## 4. Discussion

The human femur is generally described as having an anatomical anteroposterior convexity with an anterior vertex [17]. Significant effort has been made by anthropologists to quantify the femoral curvature [1–4]. Traditionally, the dimension of the femoral curvature was expressed as the subtense of the femoral chord (the absolute curvature) or the index of the curvature (the relative curvature) as measured using calipers on the lateral view [2, 3, 29]. Bruns et al. first regarded the femoral curvature as part of a virtual circle in order to manage potential complications caused by mismatches between the femur and implant [14]. Orthopedic researchers have begun to study the RFC and have attempted to incorporate this measure into the design of IM nails that are more suitable for the anatomy of the femoral canal. However, the traditional measurement methods for the curvature of the femur were performed based on the AP or lateral radiographs, and the measurement methods previously used to calculate the RFC were inconsistent and complicated, mainly including the methods of measuring the curvatures of the femoral shaft [15, 16] or the femoral medullary canal [13, 17].  Table 4 shows the methods used to measure the RFC according to previous studies published in English by orthopedic researchers. It can be seen that, except Chantarapanich et al.'s study [24], all other researchers used the 2D measurements tools and methods, mainly including lateral radiographs and images, to measure the RFC. In this study, we constructed the femur-specific coordinate system by using the CAD software. In 3ds Max software, the femur models were simulated to be placed on the coronal (*X*-*Z*) plane (Figure 1(b)) and ensure that the distal aspects of the medial and lateral femoral condyles contacted the horizontal (*X*-*Y*) plane which was perpendicular to the coronal (*X*-*Z*) plane. Thus, the direction perpendicular to the coronal (*X*-*Z*) plane could be considered the true AP view of the femur, which could be standardized in each femoral model.

Egol et al. determined the RFCs of 948 femurs by measuring the exterior of the femur on lateral digital images [15]. However, Zirkle suggested that the accuracy of the Egol method could be improved by selecting points from cross-sectional imaging of the femur. Stephenson and Seedhom investigated the geometries of the femoral medulla and observed that the thickness of the femoral anterior cortex was different from the posterior counterpart and that the femoral medulla did not lie centrally within the femur but slightly anteriorly [30]. This latter observation was supported by Buford Jr. et al. [19], who demonstrated that the curvatures of the exterior femur and medullary canal were not equivalent. In Buford Jr. et al.'s study, the authors investigated the difference between sagittal RFCs of the anterior cortex and medullary canal of 3D models and reported the presence of three femur subtypes in which the RFC of the anterior cortex might be greater than, equivalent to, or smaller than the RFC of the medullary canal.

For any implant to be located in the canal of the femur, it is vitally important that an in-depth understanding of the canal is obtained; thus, the RFC calculated based on the anatomy of the medullary canal might be more informative to clinical strategies. Harper and Carson established a formula to measure the curvature of the medullary canal on lateral radiography [17]. To examine the importance of femoral sagittal bowing on TKA, Tang et al. divided the curvature of the medullary canal into three parts and measured each individual RFC on lateral radiography, and the results showed that the radius of the distal third curvature was the smallest, followed by the middle third and proximal third [13]. Using a similar technique, Lu et al. also simulated digital lateral radiographs using 3D femoral models; however, these authors extracted the centerline from the exterior of the femur rather than the medullary canal, resulting in different findings that the curvature of the distal third was the greatest, followed by the proximal third and middle third [6].

Maratt et al. reconstructed the 3D models of femurs obtained from 1,961 patients and subsequently calculated the RFCs from the inner anterior cortex of the femur and the medullary canal on the simulated lateral radiographs, respectively [18]. The authors analyzed the potential effect of the general characteristics of the patients on the anatomical parameters of the femur and concluded that the femoral length was positively correlated with the RFC, with Pearson's *r* of 0.38. In addition to previous studies of the femoral anterior curvature, some investigators have also emphasized the existence of femoral coronal curvature, including medial or lateral bowing, and its clinical implications [20–23].

With regard to the various measurement methods and contrasting descriptions and definitions used to assess femoral curvature, important characteristics of the femur may be ignored, which could reflect accurate space information regarding the femoral curvature. To the best of our knowledge, only Chantarapanich et al. used 3D measurement techniques to analyze the femoral curvature in 3D space [24]. In their study, the authors fitted the centerline of the 3D femoral canal and its projection along the sagittal and coronal planes into three circles in 3D space and calculated and analyzed the correlations among the corresponding radii.

In the present study, after the centerline of the femoral canal was fitted into a circle, we proposed the aviation term “banking angle” to describe the intersection angle between the femoral curvature plane and coronal plane. Our results showed that the banking angle ranged from 61.9 to 139.4 degrees. When the banking angle was less than 90 degrees, the radius of the coronal projection of the 3D femoral curvature would be increased with an increase in banking angle; when the banking angle was greater than 90 degrees, the radius of the coronal curvature would be decreased with an increase in banking angle. This nonlinear relationship between the banking angle and coronal curvature might explain the results obtained by Chantarapanich et al.'s study, which showed that the 3D RFC was closely correlated with the sagittal RFC but not the coronal RFC. The wide range of the banking angle was approximately 80 degrees (61.9–139.4 degrees) in the present study, which may also explain the results obtained from the 3 section methods in the study performed by Tang et al. The reason why the radius of the distal third curvature was the smallest, followed by the middle third and proximal third, might be because most patients included in Tang et al.'s study showed banking angles that were greater than 90 degrees.

Furthermore, to investigate the factors influencing the RFC and banking angle, stepwise linear regression models were performed. These results showed that the femoral length and gender were the most relevant factors in determining the RFC and banking angle, respectively. This correlation between the femoral length and RFC was similar to that reported in the study by Maratt et al [18].

The advantage of the current study was that the methods used to standardize the position of the femoral models in 3D space and the objective methods used to extract the anatomical parameters of the femur were automated. However, this study had some limitations. First, the radii of sagittal RFCs were not calculated in all femurs; however, their relationship with the 3D RFC may be sufficiently explained by the banking angle analysis shown in Figures 3 and 4. Second, due to limitations in space and scope, we did not incorporate the anatomical parameters of the proximal femur, such as the femoral neck anteversion, and reveal their potential correlations with the diaphyseal characteristics of femurs in this study. Third, as a new term, the banking angle of the femur has not been proposed by previous researchers. So it was difficult to predict how ignoring banking angel of femur in calculating RFC would have some clinical impact. More further clinical studies are needed to reveal the potential clinical impact of the banking angle of the femur. Fourth, due to different measurement basis, it was difficult to incorporate the findings of the paper into a method to assess femoral curvature in plain radiography. However, further CAD researches might be performed to extract the 3D morphological parameters of the femurs from the plain radiography and assess the femoral curvature in plain radiography.

## 5. Conclusion

This study concluded that the banking angle of the femur was significantly larger in the female than in male and should not be ignored when the femoral curvature was studied and measured. Furthermore, this study established the methodological basis and anatomical parameters for the development of suitable femoral IM implants for elderly Chinese people.

## Supplementary Material

The simulation experiment to verify the interaction between the torus and inner femoral cortex. The axial plane was perpendicular to plane A and B in Figure 2 simultaneously, which could move alone the centerline of the canal. As shown, there was no interaction between the torus and the inner femoral cortex.

## Figures and Tables

**Table 1:** The characteristics of the population and the anatomical parameters of the femur.

Items	Male (*n* = 294)		Female (*n* = 132)		*t*-test		Total (*n* = 426)	
	Mean ± SD	Range	Mean ± SD	Range	*t*	*p*	Mean ± SD	Range
Age (years)	64.49 ± 12.86	15–85	69.68 ± 8.39	50–85	—	<0.001^△^	66.10 ± 11.89	15–85
Height (m)	1.69 ± 0.058	1.48–1.84	1.58 ± 0.061	1.40–1.77	17.95	<0.001	1.66 ± 0.078	1.40–1.84
Weight (kg)	68.58 ± 9.77	43–97	62.03 ± 9.9	37–86	6.38	<0.001	66.66 ± 10.3	37–97
Length (mm)	438.04 ± 19.6	386–485	405.86 ± 22.61	352–487	14.93	<0.001	428.07 ± 25.38	352–487
RFC (mm)	1015.72 ± 212.19	616–2029	872.81 ± 174.57	511–1375	6.78	<0.001	971.44 ± 211.68	511–2029
BA^*∗*^ (degrees)	91.21 ± 10.81	61.9–120.5	98.52 ± 12.85	64.1–139.4	−6.07	<0.001	93.48 ± 11.95	61.9–139.4
Diameter (mm)	10.68 ± 1.4	6.60–16.20	10.1 ± 1.78	6.00–14.60	—	<0.001^△^	10.50 ± 1.55	6.00–16.20

^△^The data of the age and the minimum diameter of the canal were nonnormal distribution and tested using the Manny-Whitney *U* test. ^*∗*^The BA represented the banking angle; diameter represented the isthmic diameter; length represented the femoral length.

**Table 2:** Paired samples *t*-test of the anatomical measurements of the femur.

Items	Right (*n* = 213, mean ± SD, mm)	Left (*n* = 213, mean ± SD, mm)	Difference (*n* = 213, mean ± SD, mm)	*p*
Length (mm)	427.77 ± 25.53	428.36 ± 25.29	−0.59 ± 3.67	<0.001
RFC (mm)	943.15 ± 197.36	999.73 ± 221.98	−56.58 ± 96.05	<0.001
BA^*∗*^ (degrees)	93.09 ± 11.52	93.86 ± 12.39	−0.77 ± 7.68	0.145
Diameter (mm)	10.41 ± 1.56	10.59 ± 1.53	−0.19 ± 0.61	<0.001

^*∗*^The BA represented the banking angle; diameter represented the isthmic diameter; length represented the femoral length.

**Table 3:** The correlation analysis among the characteristics of the population and the anatomical parameters of the femur.

Pearson's *r*	Age	Height	Weight	Diameter	RFC	BA^*∗*^	Length
Age	1	−0.356	−0.378	−0.013	−0.090	0.244	−0.304
Height	*p* < 0.001	1	0.549	0.206	0.339	−0.248	0.845
Weight	*p* < 0.001	*p* < 0.001	1	0.104	0.108	−0.038	0.437
Diameter	*p* = 0.795	*p* < 0.001	*p* = 0.032	1	0.012	0.002	0.235
RFC	*p* = 0.063	*p* < 0.001	*p* = 0.026	*p* = 0.805	1	−0.249	0.369
BA	*p* < 0.001	*p* < 0.001	*p* = 0.434	*p* = 0.969	*p* < 0.001	1	−0.223
Length	*p* < 0.001	*p* < 0.001	*p* < 0.001	*p* < 0.001	*p* < 0.001	*p* < 0.001	1

^*∗*^The BA represented the banking angle; diameter represented the isthmic diameter; length represented the femoral length.

**Table 4:** Previous measurements of the RFC by orthopaedic researchers published in English.

Author	Year	Number	Subject	Origin	RFC (mean ± SD, mm)	Methods
Onoue et al. [16]	1979	160	Patient	Japanese	The femoral shaft: 1159 ± 122	Lateral radiograph

Harper and Carson [17]	1987	14	Cadaver	NA	1144	Lateral radiograph

Egol et al. [15]	2004	948	Cadaver	White, black	The exterior of the femur: 1200 ± 360	Lateral image

Tang et al. [13]	2005	100	Patient	Chinese	(OA versus RA) Proximal: 1099.8 ± 237.4 versus 978.6 ± 88.4; middle: 935.2 ± 122.8 versus 874.3 ± 60; distal: 722.3 ± 77.8 versus 673.6 ± 58.8	Lateral radiograph

Chantarapanich et al. [24]	2008	99	Cadaver	Thai	3D RFC: 895.46 ± 238.06; sagittal RFC: 891.46 ± 234.87	3D reconstruction

Wang et al. [27]	2009	18	Cadaver	Chinese	888.89 ± 160.47	Cast mold of canal

Lu et al. [6]	2012	73	Patient	Chinese	The exterior of the femur: proximal: 769.5^*∗*^; middle: 1537.25^*∗*^; distal: 622.45^*∗*^	Lateral radiograph

Maratt et al. [18]	2014	3922	Cadaver	Mixed	The medullary: 1120 ± 260; the inner anterior cortex: 1450 ± 550	Lateral radiograph

Buford et al. [19]	2014	19	Cadaver	White	Anterior cortex: 1446 ± 397	Lateral radiograph

NA: nonapplicable.

*∗*: calcutaed from the curvatures in the reference.
